# Risk Assessment and Hierarchical Risk Management of Enterprises in Chemical Industrial Parks Based on Catastrophe Theory

**DOI:** 10.3390/ijerph9124386

**Published:** 2012-12-03

**Authors:** Yu Chen, Guobao Song, Fenglin Yang, Shushen Zhang, Yun Zhang, Zhenyu Liu

**Affiliations:** Key Laboratory of Industrial Ecology and Environmental Engineering (MOE), School of Environmental Science and Technology, Dalian University of Technology, Dalian 116024, China; Email: chenyu@dlut.edu.cn (Y.C.); gb.song@dlut.edu.cn (G.S.); zhangss@dlut.edu.cn (S.Z.); zhangyun@dlut.edu.cn (Y.Z.); lzhenyu@yeah.net (Z.L.)

**Keywords:** risk assessment and management, catastrophe theory, chemical enterprises, index system

## Abstract

According to risk systems theory and the characteristics of the chemical industry, an index system was established for risk assessment of enterprises in chemical industrial parks (CIPs) based on the inherent risk of the source, effectiveness of the prevention and control mechanism, and vulnerability of the receptor. A comprehensive risk assessment method based on catastrophe theory was then proposed and used to analyze the risk levels of ten major chemical enterprises in the Songmu Island CIP, China. According to the principle of equal distribution function, the chemical enterprise risk level was divided into the following five levels: 1.0 (very safe), 0.8 (safe), 0.6 (generally recognized as safe, GRAS), 0.4 (unsafe), 0.2 (very unsafe). The results revealed five enterprises (50%) with an unsafe risk level, and another five enterprises (50%) at the generally recognized as safe risk level. This method solves the multi-objective evaluation and decision-making problem. Additionally, this method involves simple calculations and provides an effective technique for risk assessment and hierarchical risk management of enterprises in CIPs.

## 1. Introduction

In recent years, chemical industrial parks (CIPs) have become one of the mainstreams of international development and a new developmental model in the Chinese chemical industry. CIPs can improve the comprehensive utilization of raw materials and energy through intensification of production units, mutual supply of raw materials among units, and unified planning, development and construction of relevant infrastructure, logistical facilities, waste centralized treatment facilities, transportation and info-communication, further reducing the construction costs of relevant projects in CIPs and maximizing the economic and environmental benefits. While CIP construction promotes development of the regional economy and chemical industry, it also poses new safety issues and environmental risks [1,2,3]. Presently, a core issue of concern to those who construct and manage CIPs is the effective prevention and mitigation of losses and impacts caused by risk accidents, and implementation of effective risk management that can ensure safe production and social stability to enable sound development of the enterprises.

**Table 1:** Risk assessment method of the chemical industry.

Method	Study (year)	Detial
Material risk analysis approach	Cave *et al*. (1997) [4]	Defined the environmental hazard index (EHI) and applied it to the chemical industry for a case study of the degree of environmental impacts.
	Gunasekera and Edwards (2003) [5]	Employed the atmospheric hazard index (AHI) for comparative analysis of environmental risks of the same chemical products.
production process safety assessment approach	Shah *et al*. (2003) [6]	Constructed a hierarchy of safety, health and environment, which involves multiple layers of material, reaction, devices, and safety technology for comparison of inherent risks in different production processes.
	Faisal *et al*. (2005) [7]	Proposed a comprehensive inherent safety index evaluation method that considers the economic evaluation and risk assessment for evaluation of the inherent level of risk associated with production processes.
	Wei *et al*. (2008) [8]	Suggested the use of a layer of protection analysis (LOPA) and applied it to risk assessment of chemical reactions during hydroxylamine production.
Environmental risk index method	Achour *et al*. (2005) [9]	Established the material quantitative index of process flow and the inherent risk index of risk source for environmental risk assessment of production processes.
	Jia *et al*. (2010) [10]	Constructed a comprehensive evaluation index of accidental environmental risks from petrochemical enterprises based on the hazardous materials present, production processes, and enterprise distribution.
	EU (1993) [11] (2003) [12]	Applyed a comprehensive risk assessment approach that considers environmental and human exposure and evaluates the risks caused by the exposure.
	Huang *et al*. (2011) [13]	Established a CIP risk assessment index system that considers risk at both the level of the CIP and relevant enterprises, and then conducted a case study in Jiangsu Province, China.

Considering the characteristics of enterprises in CIPs, which generally include multiple types of businesses, complex production processes, and large stocks of hazardous chemicals, several researchers have conducted enterprise risk assessment of the chemical industry while focusing on the risk associated with materials, safety of production processes, and indices of environmental risks (Table 1). Among these, the material risk analysis approach describes enterprise environmental risks by calculating the degree of environmental impacts of chemicals in use. The production process safety assessment approach analyzes and compares the risks associated with the same chemical products used in different production processes, or risks at different steps of the same production process. The environmental risk index method characterizes enterprise risks via the analysis of a series of assessment indices. Overall, the existing approaches for risk assessment primarily focus on specific risk aspects, such as the production process and/or storage sites of hazardous materials (e.g., tank fields). However, few methods are currently available for comprehensive risk assessment, and in those that are available, index weighting is affected by subjective factors, further influencing the results of such assessments.

The environmental risk system of CIPs includes the risk associated with the source, control mechanisms and risk receptors. In other words, the degree of risk is not only dependent on the inherent risk size of the risk source, but is also related to the prevention and control level and the degree of environmental sensitivity of the receptor. If an enterprise has strong prevention and emergency response capabilities for sudden risk accidents, its risk level should be reduced appropriately. Similarly, if an environmentally sensitive receptor adjacent to the enterprise has a low degree of sensitivity, the associated risk level should be reduced appropriately. Therefore, systematic analysis of risks is the key to risk management. Based on these, we define comprehensive risk level as an integrated indicator that considers the inherent risk of source, the prevention and control level, and the degree of environmental sensitivity of the receptor. Due to the large number and wide distribution of enterprises in CIPs and difficulties associated with risk management, research regarding the comprehensive risk assessment approach for relevant enterprises is the premise and foundation of CIP risk management. In this study, an risk assessment index system was established according to the risk system theory to characterize the comprehensive risk level of the enterprises in a CIP based on three indices, the inherent risk associated with the risk source, effectiveness of prevention and control mechanisms, and vulnerability of receptors.

Catastrophe theory is a branch of dynamical systems theory that investigates discontinuous changes and catastrophes [14]. Catastrophe theory can deal directly with discontinuity without linking any specific internal mechanisms; therefore, it is particularly applicable to studies of systems with unknown internal functions. In recent years, catastrophe theory has been widely employed in multi-index comprehensive assessment studies [15,16,17]. In this study, an index system for risk assessment of chemical enterprises based on catastrophe theory was established. The initial fuzzy membership function and normalized formula were employed for quantitative assessment via the recursive algorithm. The obtained total catastrophe membership values of risks associated with enterprises were used to determine their risk levels. The results provide a basis for decision making in risk management of CIPs.

## 2. Methods

### 2.1. Catastrophe Theory

Risk discussed in this study refers to environmental pollution accidents. It means any occurrence including a major emission, fire or explosion involving one or more hazardous chemicals and resulting from some uncontrolled development in the course of industrial activity or storage or due to transportation accidents leading to serious effects, cause loss of life and property, including adverse effects on the environment. Risks occurring due to natural disasters, war and terrorism are not a matter to be discussed in this paper. The occurrence of an environmental pollution accident can be interpreted as a qualitative change in the system caused by changes in internal parameters. The shift of the system from a secure to an accident state is a catastrophic event. The application of catastrophe theory to accident prevention management of chemical plants plays an important role in relevant accident prevention.

Catastrophe theory uses mathematical tools to describe the system parameter regions in a stable or unstable states and the parameter range of system catastrophes for mathematical modeling of the catastrophe process. Catastrophe models are characterized by the classification of critical points according to the system potential function, and the induction of several elementary catastrophe models according to the characteristics of discontinuous changing states near critical points. The folded catastrophe, cusp catastrophe, swallowtail catastrophe and butterfly catastrophe models are commonly used [18]. In these models, *f*(*x*) represents the potential function of system state variables, and the coefficients of the state variables *x*, *i.e.*, *a*, *b*, *c*, *d*, represent its control variables. The state and control variables of system potential function are contradictory, and various control variables interplay to form contradictions. Consequently, any state of the system is a function of state and control variables. The set of all critical points of the potential function *f*(*x*) forms an equilibrium surface, and its equation is derived from the first derivative of *f*(*x*), namely, *f*′(*x*) = 0. An associated singularity set is derived from the second derivative of *f*(*x*), namely, *f*″(*x*) = 0. The bifurcation set equation of the catastrophe system is obtained through elimination of *x* from *f*′(*x*) = 0 and *f*″(*x*) = 0. When various control variables in the bifurcation set equation meet the requirements, a catastrophe will occur in the system. The normalized formula is derived from the bifurcation set equation in a decomposed form and then used to transform different qualitative states of various variables into the same qualitative state, which is represented by the state variable. The normalized formula, which can be further used to derive the total catastrophe membership value that characterizes the system state, is the basic formula used for comprehensive analysis and assessment based on catastrophe theory (Table 2).

**Table 2:** Summary of catastrophe models.

Category	Potential function	Bifurcation set	Normalization formula	Dimension of control variables
Fold model	*f*(*x*) = *x*^3^ + *ax*	*a* = −3*x*^2^	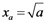	1
Cusp model	*f*(*x*) = *x*^4^ + *ax*^2^ + *bx*	*a* = −6*x*^2^, *b* = 8*x*^3^	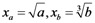	2
Swallowtail model	*f*(*x*) = *x*^5^ + *ax*^3^ + *bx*^2^ + *cx*	*a* = −6*x*^2^, *b* = 8*x*^3^	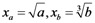	3
Butterfly model	*f*(*x*) = *x*^6^ + *ax*^4^ + *bx*^3^ + *cx*^2^ + *dx*	*a* = −10*x*^2^, *b* = 20*x*^3^	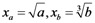	4

### 2.2. Establishment of A Catastrophe Model for Accidental Risk Assessment of Chemical Enterprises

#### 2.2.1. Index System Establishment

According to environmental risk systems theory, an environmental risk system consists of a risk source, primary control mechanism, secondary control mechanism and risk receptor [19]. The primary control system refers to facilities controlling risk source and human-related factors of maintenance and management, whereas the secondary control system refers to control of natural conditions of transmission risk. In this study, both primary and secondary control mechanisms were considered:

(1) Selection of goal layer A (first-level index)

Layer A is the highest level of the index with a single index value. We considered the environmental risk assessment index of chemical enterprises in CIP to be layer A.

(2) Selection of criteria layer B (second-level index)

According to environmental risk systems theory, layer B consists of three indices, the inherent risk associated with the risk source, effectiveness of prevention and control mechanisms, and vulnerability of the receptors.

(3) Selection of sub-criteria layer C (third-level index)

The source of risk, which forms the main body of an accidental risk, includes the inherent toxicity and flammability/explosibility of risk factors, as well as the possibility of sudden risk accidents and the operating condition of production processes [10]. Therefore, the sub-criteria layer of the inherent risk associated with the risk source includes two sub-indices, the risk factor essential characteristic index and the risk accident characteristic index. The effectiveness index of the prevention and control mechanism includes the environmental management mechanism index and considers the positive role that fire departments and medical institutions play in emergency rescue [20]. The risk receptors include human and other environmentally sensitive receptors vulnerable to risk accidents [21]. When evaluating risk associated with an accident, human beings are the primary objects of protection, followed by an environment that ensures the quality of human life, and finally the property that relates to human production and living. In this study, we focused on the indices of population- and environmentally-sensitive receptors. 

(4) Selection of alternative layer *x* (fourth-level index)

The presence of a risk factor is a prerequisite for the occurrence of any risk event. Major risk characteristics of chemical enterprises include toxic and hazardous risk materials in storage and in use during production, flammable and explosive hazardous materials that cause secondary pollution, and chemically reactive hazardous materials. Because the quantity of hazardous materials forms the basis for determining whether these materials form a major risk source, the risk factor index should also include a quantitative hazardous material sub-index. The accident hazard characteristic index includes the probability of risk accident and the operating pressure and temperature conditions of production processes. The main pathway to reduction of regional environmental risks is increased regulation of major CIP environmental risk sources, and this index includes the risk manager of CIPs, the command center for early warning and emergency response, completeness of contingency plans, and real-time monitoring of each enterprise. The fire and rescue status index reflects the emergency response capability of fire departments post-accident and includes the arrival time of the fire department at the site of the accident and the quantity of emergency facilities. Medical rescue ensures that wounded are out of danger, and this index includes the medical rescue time and equipped facilities. The most direct victims of an risk accident are sensitive populations surrounding the chemical enterprises, and the population sensitive receptor index is related to the distance of populations from the CIP, as well as the density and degree of sensitivity of surrounding populations. These three indices are considered to be the alternative layer index of the population-sensitive receptor. The environmentally sensitive receptor is directly affected by its distance from the risk area and its degree of sensitivity, which are both considered to be the alternative layer index of the environmentally sensitive receptor.

**Figure 1:**
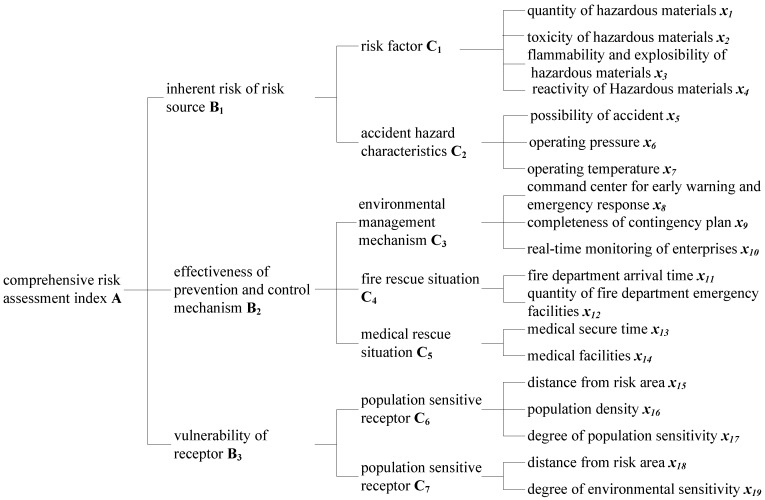
Comprehensive risk assessment index system for enterprises in chemical industrial parks.

Overall, the comprehensive risk index system of CIP enterprises consists of three indices in the criteria layer, seven indices in the sub-criteria layer and 19 indices in the alternative layer. These indices cover the major risk factors of CIP enterprises and thus reflect the environmental risk conditions of these facilities (Figure 1).

#### 2.2.2. Determination of Primary and Secondary Status of Variable Indices in Each Layer

(1) Primary and secondary status of variable indices in the criteria layer (B)

Of the three indices in the criteria layer, the inherent risk of the risk source is the source of risk events. This factor is also prerequisite for the occurrence of risk accidents and is therefore given the primary priority [19]. Effective prevention and control mechanisms are an important guarantee for timely control and risk reduction following accidents and thus have the second priority. The receptor vulnerability index has the third priority.

 (2) Primary and secondary status of variable indices in the sub-criteria layer (C)

The inherent risk indices of risk source include the risk factor index and the accident hazard characteristic index. The former directly reflects the essential characteristics of risk source and thus has priority over the latter.

A sound environmental management mechanism provides excellent warnings prior to an accident, as well as systematic emergency command at the site of an accident and coordination of fire control and medical treatment post-accident. Therefore, the environmental management mechanism index has the highest priority. Fire rescue can control the effects of an accident, thus its index has the second priority. The medical rescue index has the third priority. Of the two sub-indices of receptor vulnerability, the population-sensitive receptor index has priority over the environmentally sensitive receptor index, as human beings are the primary objects of protection.

(3) Primary and secondary status of risk indices in the alternative layer (*x*)

For chemical enterprises, there are four sub-indices of the risk factor index in the alternative layer: toxicity, quantity, flammability and explosibility, and reactivity of hazardous materials. Of these, the quantity of hazardous materials has the highest priority, as leakage can directly impact human health and environmental safety. The accident hazard characteristic index includes two sub-indices, the probability of accident and operating pressure and temperature conditions. Of these, the former directly corresponds to the consequences of an accident. A risk accident with a high probability commonly results in small consequences, whereas that with a low probability results in relatively large consequences. Therefore, accident probability has priority over operating pressure and temperature conditions. Among the three sub-indices of the environmental management mechanism index, the command center for early warning and emergency response has the highest priority, as a sound risk management department is the foundation of environmental management. Of the two sub-indices of the fire rescue index, the arrival time of the fire department has priority over the quantity of emergency facilities because arrival time is a key factor involved in rescue. Similarly, medical rescue time has priority over the quantity of medical treatment facilities in the medical rescue index because arrival time is a key factor in life-saving. Among the three sub-indices of the population-sensitive receptor index, the distance of population from the risk area has priority over population density and degree of population sensitivity because the former directly determines the degree of risk injury. Of the two sub-indices of the environmentally sensitive receptor index, the distance from risk area has priority over the degree of environmental sensitivity.

#### 2.2.3. Control Variable Standardization

The raw data of the alternative layer are standardized to obtain multi-dimensional catastrophe fuzzy membership values between 0 and 1, which solves problems related to non-comparative raw data from alternative layer indices caused by different ranges and units. All the indices are standardized using the following equations:

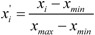
(1)

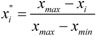
(2)
where *i* is the index, *x_i_* is the original value of *i*, *x_max_* is and *x_min_* are respectively the maximum and the minimum value of *i*. Equation (1) is for positive indices and Equation (2) is for negative indices.

As shown in Figure 1, control variables *x_1_* to *x_4_* constitute the butterfly catastrophe model C_1_, while control variables *x_5_* to *x_7_*, *x_8_* to *x_10_*, and *x_8_* to *x_10_* constitute the swallowtail catastrophe model C_2_, C_3_ and C_6_, respectively, and control variables *x_11_* to *x_12_*, *x_13_* to *x_14_*, and *x_18_* to *x_19_* constitute the cusp catastrophe model C_4_, C_5_ and C_7_, respectively. Of these, *x_1_* to *x_7_*, *x_11_*, *x_13_*, *x_15_*, *x_16_*, and *x_18_* are smaller-the-better type indices, which can be calculated using Equation (1). The control variables *x_8_* to *x_10_*, *x_12_*, *x_14_*, *x_17_* and *x_19_* are bigger-the-better type indices, which can be calculated using Equation (2). The control variables *x_2_* to *x_5_*, *x_8_* to *x_10_*, *x_12_*, *x_14_*, *x_17_*, and *x_19_* are qualitative indices, with x_2_ taken as an example for value selection and instruction. According to the Hazardous Materials Identification System for Emergency Treatment (NFPA 704) of the National Fire Protection Association (NFPA), the toxicity of hazardous materials can be classified at five levels (0–4), with level 0 indicating the least hazard to humans, and level 4 indicating the greatest hazard to humans. Relevant data were obtained by querying the list of hazardous materials. The values of other qualitative indices were selected in a similar fashion according to the literature [22,23].

#### 2.2.4. Risk Level Transformation

The comprehensive evaluation values obtained using catastrophe theory are often high, with small variations [18]. This is mainly due to the characteristics of normalized formulas used in the catastrophe evaluation method [24]. Consequently, it is difficult to directly determine the actual safety level using catastrophe-based evaluation results. The comprehensive evaluation index can generally be divided into five levels according to the principle of equal distribution function [25]. Accordingly, the chemical enterprise risk level can be divided into the following five levels: 1.0 (very safe), 0.8 (safe), 0.6 (generally recognized as safe, GRAS), 0.4 (unsafe), 0.2 (very unsafe). Given the premise of the catastrophe evaluation index system, when the membership values of the corresponding alternativelayer indices are *x*, the total comprehensive evaluation values should also be *x*. When the membership values of corresponding alternative layer indices are *x_i_* (*i* = 1,2, ..., m), associated comprehensive evaluation values can be obtained using the catastrophe evaluation method *y_i_* (*i* = 1,2, ... , m). The corresponding hierarchical relationship between *y* and *x* is shown in Table 3.

**Table 3:** Corresponding values between risk assessment results of catastrophe model and ordinary-use values at different risk levels.

Risk level	Relative degree of membership obtained by catastrophe model	Corresponding ordinary-use values
Very safe	>0.9884	>0.8
Safe	0.9884–0.9738	0.8–0.6
Generally recognized as safe (GRAS)	0.9738–0.9539	0.6–0.4
Unsafe	0.9539–0.9213	0.4–0.2
Very unsafe	<0.9213	<0.2

## 3. Examples

### 3.1. Study Site

**Figure 2:**
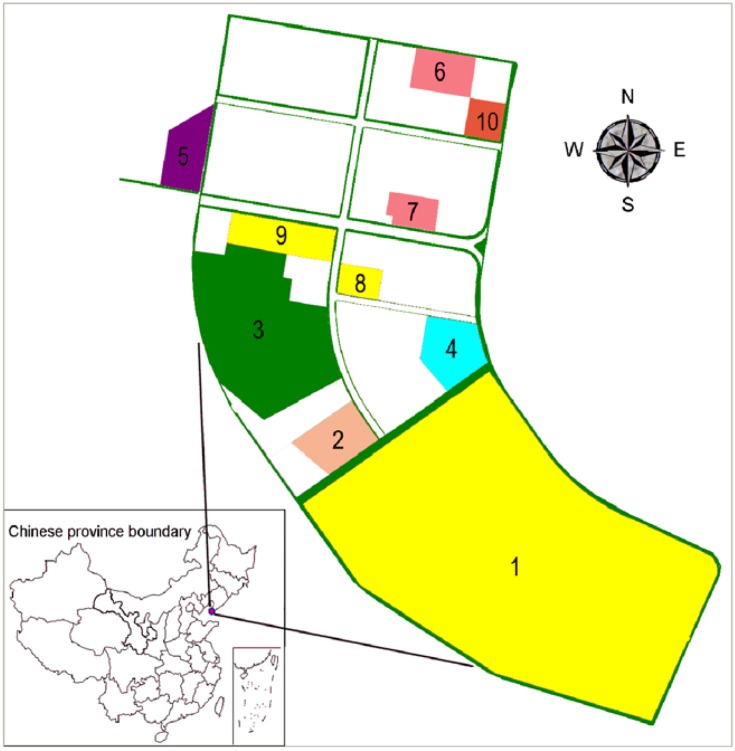
Location of the Songmu Island Chemical Industrial Park, China.

The Songmu Island CIP in China covers an area of 7.8 km^2^ and consists of syngas chemical, petrochemical, and fine chemical industries, with ten chemical enterprises (Figure 2). Among these, syngas chemical enterprises mainly produce chemical products such as ammonia and methanol, petrochemical enterprises use heavy oil to produce chemical raw materials such as ethylene and propylene, as well as oil products, and fine chemical enterprises produce dyes, paints, and pesticides (Appendix 1). Dangerous substances involved in 10 chemical enterprises are ammonia, sulfuric acid, gasoline, chlorine, MTBE, kerosene, toluene, chlorine, oleic acid and glycerin, respectively.

### 3.2. Catastrophe Theory Application

Normalization was performed according to Equations (1) and (2). The raw data of control variables were converted to comparable, dimensionless data within the range of 0–1 (Appendix 2). An example of the calculation of total catastrophe membership degree is presented in Appendix 3.

**Figure 3:**
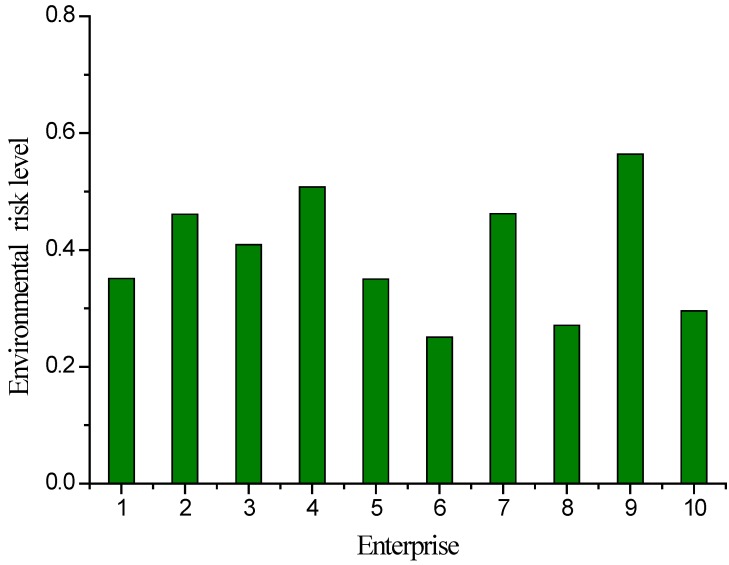
Comprehensive risk levels of chemical enterprises in Songmu Island Chemical Industrial Park.

Similarly, total catastrophe membership degree values were obtained for other chemical enterprises in the Songmu Island CIP (Table 4). The comprehensive membership values were then transformed according to Table 3 (Figure 3).

**Table 4:** Results of risk assessment of chemical enterprises in Songmu Island Chemical Industrial Park.

NO.	Goal	Criteria		Sub-criteria		NO.	Goal	Criteria		Sub-criteria	
1	A = 0.9481	B_1_	0.7256	C_1_	0.6331	6	A = 0.9322	B_1_	0.9120	C_1_	0.9075
				C_2_	0.2817					C_2_	0.6614
		B_2_	0.9961	C_3_	0.9769			B_2_	0.7874	C_3_	0.7889
				C_4_	1.0000					C_4_	0.3150
				C_5_	1.0000					C_5_	0.3969
		B_3_	0.9749	C_6_	0.9710			B_3_	0.7106	C_6_	0.3333
				C_7_	0.8969					C_7_	0.6010
2	A = 0.9612	B_1_	0.8324	C_1_	0.6686	7	A = 0.9613	B_1_	0.9211	C_1_	0.7093
				C_2_	0.6080					C_2_	1.0000
		B_2_	0.9239	C_3_	0.8272			B_2_	0.8706	C_3_	0.7889
				C_4_	0.8078					C_4_	0.5650
				C_5_	0.7504					C_5_	0.6469
		B_3_	0.9890	C_6_	0.9564			B_3_	0.8823	C_6_	0.8476
				C_7_	1.0000					C_7_	0.6010
3	A = 0.9554	B_1_	0.8436	C_1_	0.7498	8	A = 0.9357	B_1_	0.7797	C_1_	0.7500
				C_2_	0.5539					C_2_	0.3333
		B_2_	0.9605	C_3_	0.9024			B_2_	0.8777	C_3_	0.8272
				C_4_	0.8873					C_4_	0.5650
				C_5_	0.8873					C_5_	0.6469
		B_3_	0.8536	C_6_	0.5003			B_3_	0.8735	C_6_	0.8155
				C_7_	1.0000					C_7_	0.2041
4	A = 0.9659	B_1_	0.8531	C_1_	0.7500	9	A = 0.9710	B_1_	0.9636	C_1_	0.8595
				C_2_	0.5932					C_2_	1.0000
		B_2_	0.9399	C_3_	0.8272			B_2_	0.9163	C_3_	0.8272
				C_4_	0.8299					C_4_	0.7504
				C_5_	0.8873					C_5_	0.7504
		B_3_	0.9785	C_6_	0.9855			B_3_	0.8499	C_6_	0.9312
				C_7_	0.8969					C_7_	0.0000
5	A = 0.9479	B_1_	0.8519	C_1_	0.7175	10	A = 0.9400	B_1_	0.9260	C_1_	0.7259
				C_2_	0.6288					C_2_	1.0000
		B_2_	0.8788	C_3_	0.8336			B_2_	0.8968	C_3_	0.8272
				C_4_	0.5650					C_4_	0.6469
				C_5_	0.6469					C_5_	0.7043
		B_3_	0.8596	C_6_	0.7662			B_3_	0.6370	C_6_	0.4694
				C_7_	0.6010					C_7_	0.2041

The results of catastrophe-based assessment showed that the comprehensive risk levels of all ten chemical enterprises in the Songmu Island CIP were below the GRAS level (Table 4, Figure 3). Specifically, five enterprises were at the GRAS level, while the remaining five were at the unsafe level. Overall, Enterprise 9 has the lowest risk. This was because the risk substance of Enterprise 9, oleic acid, has relatively low toxicity and flammability/explosibility without reactivity, and the associated operating conditions are normal temperature and pressure. Furthermore, the probability of dangerous accidents is low and the distance from sensitive sites is far. However, the total quantity of risk substances is relatively high in Enterprise 9, and its real-time monitoring capability needs to be improved. Enterprise 4 is also far from sensitive sites and has a low degree of population sensitivity. Its associated hazardous substance liquid, chlorine, has no flammability, explosibility or reactivity, but has high toxicity and probability of risk accident. Therefore, the comprehensive risk level of Enterprise 4 is second to Enterprise 2. In addition, Enterprise 6 is closest to environmentally sensitive sites, has the highest population density and the poorest emergency response capabilities. Accordingly, the comprehensive risk level of Enterprise 6 was lowest among the ten assessed enterprises and should be the focus of regional environmental risk management.

**Figure 4:**
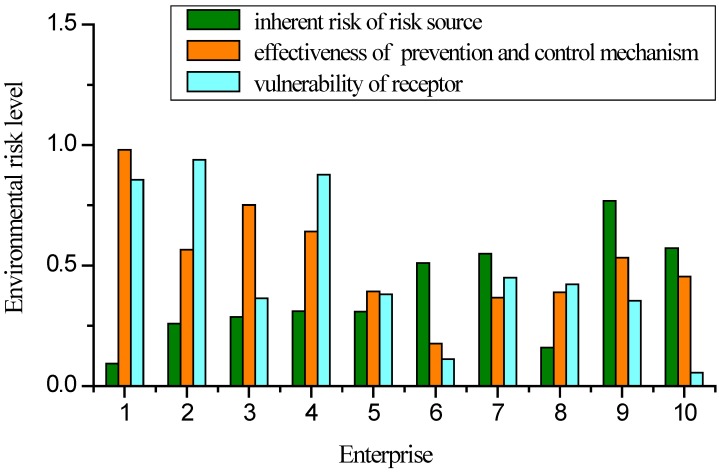
Risk levels of layer B indices of chemical enterprises in Songmu Island Chemical Industrial Park.

Figure 4 shows the risk levels of the ten CIP enterprises according to the indices of inherent risk associated with the risk source, effectiveness of prevention and control mechanisms, and vulnerability of receptors. Enterprise 9 had normal temperature and pressure operating conditions and a small probability of risk accident, with low toxicity, flammability/explosibility and reactivity of its risk substance, oleic acid (maximum safety level of inherent risk = 0.768). Other enterprises had larger inherent risks, particularly Enterprise 1, which is a large ammonia production enterprise. Enterprise 1 is characterized by a large quantity of risk substances with high toxicity, as well as a high probability of accident and high temperature and pressure operating conditions, resulting in a low inherent risk source index value of 0.094. Enterprise 1 is a large state-owned enterprise with a sound environmental management system and good fire-fighting and medical facilities; accordingly, its effectiveness index value for its prevention and control mechanisms is highest among the enterprises (0.981), which is at the very safe level. Comparatively, Enterprise 3 has a smaller effectiveness index value for its prevention and control mechanism due to the poor real-time monitoring capability. Enterprise 10 was closer to population sensitive receptor with a higher population density than other enterprises, and the surrounding environmentally sensitive sites were environmentally improved areas with a high degree of environmental sensitivity. Therefore, Enterprise 10 had the lowest index value for receptor vulnerability of 0.055, which was at the very unsafe level. By comparison, the Enterprise 2 was far from both the population and environmentally sensitive receptors, and the associated environmentally sensitive sites were areas already affected by pollution. Thus, its safety level of receptor vulnerability was 0.939, at the very safe level. The assessment results were consistent with the actual situation of these enterprises, suggesting that the evaluation method based on catastrophe theory is valid.

The catastrophe-based risk assessment of chemical enterprises in Songmu Island CIP, China showed that Enterprise 1 had largest inherent risk associated with risk source, but its associated effectiveness of prevention and control mechanism was very good and could therefore provide timely and effective risk control when a risk accident occurs. Moreover, Enterprise 1 had low receptor vulnerability and distant sensitive targets. Overall, the comprehensive risk level of Enterprise 1 ranked 5th among the ten enterprises. By comparison, Enterprise 6 had small inherent risks associated with risk source and ranked 7th among the ten enterprises. From the perspective of inherent risk index, Enterprise 6 may not be the focus of regional environmental risk management. However, due to the poor effectiveness of prevention and control mechanisms and the high vulnerability of the sensitive receptors, Enterprise 6 had a high comprehensive risk level and should be the focus of regional environmental risk management.

## 4. Conclusions

(1) Based on the environmental risk systems theory, a comprehensive evaluation index system was established for risk assessment of chemical enterprises in CIPs. The three indices included the inherent risk associated with risk source, effectiveness of prevention and control mechanisms, and vulnerability of receptors. Among these, the inherent risk index associated with risk source is determined by multiple factors such as the stock, toxicity, flammability/explosibility and production process of hazardous substances, while the effectiveness of prevention and control mechanisms is determined by the environmental management level and fire/medical rescue situation of the enterprise and the receptor vulnerability is calculated based on the density and structure of surrounding populations and the distance of sensitive targets from the enterprise.

(2) The application of catastrophe theory in comprehensive risk assessment of chemical enterprises in CIPs did not require the subjective weighting of indices, enabling transformation of the multi-objective problem into a single index for assessment, thus effectively solving the multi-objective evaluation and decision-making problem. The calculation procedure does not require a high level of technical expertise to determine the membership degree, making the calculation simple and operational. But this assessment method relies on the relative importance of indices and such process can not completely avoid human subjectivity.

(3) Catastrophe-based risk assessment of ten CIP enterprises was conducted taking the Songmu Island CIP in China as an example. The results indicated that five enterprises were at the unsafe risk level, and five were at the GRAS level. These results suggest that the overall risk associated with chemical enterprises in the Songmu Island CIP is high. The assessment results were consistent with the actual situation of these enterprises, suggesting that the evaluation method based on catastrophe theory is valid. Our assessment results reflect the risk variations in CIP enterprises and screening for key enterprises for risk management based on quantitative results of risk levels, further providing a basis for decision making in risk management. 

## Appendix

**Appendix 1:** Raw data describing the environmental risk assessment index system for chemical enterprises in Songmu Island Chemical Industrial Park.

Criteria	Sub-criteria	Alternative	1	2	3	4	5	6	7	8	9	10
B_1_	C_1_	*x_1_* (t)	351	105	5.69	0.5	4.56	20	200	0.5	2,000	2,800
		*x_5_*	3	3	1	4	1	2	2	4	1	1
		*x_3_*	1	0	3	0	3	2	3	0	1	1
		*x_4_*	2	2	0	0	1	0	0	0	0	0
	C_2_	*x_5_* (10^–6^/y)	5	1	5	5	5	5	0.5	0.5	0.5	0.5
		*x_6_* (bar)	20	7	13.5	11	5	1	1	20	1	1
		*x_7_* (°C)	500	1,000	160	20	160	80	20	20	20	20
B_2_	C_3_	*x_8_*	1	0.75	1	0.75	1	0.75	0.75	0.75	0.75	0.75
		*x_9_*	1	0.75	1	0.75	0.5	0.5	0.5	0.75	0.75	0.75
		*x_10_*	0.75	0.25	0.25	0.25	0.25	0.25	0.25	0.25	0.25	0.25
	C_4_	*x_11_* (min)	10	20	15	15	25	30	25	25	20	25
		*x_12_*	1	0.75	0.75	0.5	0.25	0.25	0.25	0.25	0.5	0.5
	C_5_	*x_13_* (min)	10	15	10	10	15	10	10	10	15	10
		*x_14_*	1	0.5	0.75	0.75	0.5	0.5	0.5	0.5	0.5	0.75
B_3_	C_6_	*x_15_* (m)	1,000	1,000	800	1,100	700	500	1,000	900	1,100	600
		*x_16_* (person/hm^2^)	20	25	40	25	45	60	50	50	40	60
		*x_17_*	3	3	3	3	3	3	3	3	3	3
	C_7_	*x_18_* (m)	100	100	100	100	50	50	50	50	40	50
		*x_19_*	4	5	5	4	4	4	4	3	3	3

**Appendix 2:** Catastrophe fuzzy membership functions of the risk assessment indices for chemical enterprises in Songmu Island Chemical Industrial Park.

Criteria	Sub-criteria	Alternative	1	2	3	4	5	6	7	8	9	10
B_1_	C_1_	*x_1_*	0.8748	0.9627	0.9981	1.0000	0.9985	0.9930	0.9287	1.0000	0.2858	0.0000
		*x_2_*	0.3333	0.3333	1.0000	0.0000	1.0000	0.6667	0.6667	0.0000	1.0000	1.0000
		*x_3_*	0.6667	1.0000	0.0000	1.0000	0.0000	0.3333	0.0000	1.0000	0.6667	0.6667
		*x_4_*	0.0000	0.0000	1.0000	1.0000	0.5000	1.0000	1.0000	1.0000	1.0000	1.0000
	C_2_	*x_5_*	0.0000	0.8889	0.0000	0.0000	0.0000	0.0000	1.0000	0.0000	1.0000	1.0000
		*x_6_*	0.0000	0.6842	0.3421	0.4737	0.7895	1.0000	1.0000	0.0000	1.0000	1.0000
		*x_7_*	0.5102	0.0000	0.8571	1.0000	0.8571	0.9388	1.0000	1.0000	1.0000	1.0000
B_2_	C_3_	*x_8_*	1.0000	0.7500	1.0000	0.7500	1.0000	0.7500	0.7500	0.7500	0.7500	0.7500
		*x_9_*	1.0000	0.7500	1.0000	0.7500	0.5000	0.5000	0.5000	0.7500	0.7500	0.7500
		*x_10_*	0.7500	0.2500	0.2500	0.2500	0.2500	0.2500	0.2500	0.2500	0.2500	0.2500
	C_4_	*x_11_*	1.0000	0.5000	0.7500	0.7500	0.2500	0.0000	0.2500	0.2500	0.5000	0.2500
		*x_12_*	1.0000	0.7500	0.7500	0.5000	0.2500	0.2500	0.2500	0.2500	0.5000	0.5000
	C_5_	*x_13_*	1.0000	0.5000	0.7500	0.7500	0.2500	0.0000	0.2500	0.2500	0.5000	0.2500
		*x_14_*	1.0000	0.5000	0.7500	0.7500	0.5000	0.5000	0.5000	0.5000	0.5000	0.7500
B_3_	C_6_	*x_15_*	0.8333	0.8333	0.5000	1.0000	0.3333	0.0000	0.8333	0.6667	1.0000	0.1667
		*x_16_*	1.0000	0.8750	0.5000	0.8750	0.3750	0.0000	0.2500	0.2500	0.5000	0.0000
		*x_17_*	1.0000	1.0000	0.0000	1.0000	1.0000	1.0000	1.0000	1.0000	1.0000	1.0000
	C_7_	*x_18_*	1.0000	1.0000	1.0000	1.0000	0.1667	0.1667	0.1667	0.1667	0.0000	0.1667
		*x_19_*	0.5000	1.0000	1.0000	0.5000	0.5000	0.5000	0.5000	0.5000	0.5000	0.0000

### Appendix 3. An Example of the calculation of total catastrophe membership degree.

Here, Enterprise 5 was taken as an example to show the calculation of total catastrophe membership degree.

#### Calculation of C-level Membership Degree Using x as the Control Variable

C_1_ and *x* constitute a butterfly catastrophe model

C_2_ and *x* constitute a swallowtail catastrophe model

C_3_ and *x* constitute a swallowtail catastrophe model

C_4_ and *x* constitute a cusp catastrophe model

C_5_ and *x* constitute a cusp catastrophe model

C_6_ and *x* constitute a swallowtail catastrophe model

C_7_ and *x* constitute a cusp catastrophe model

#### Calculation of B-level Membership Degree Using C-level Indices as Control Variables

B_1_ and C constitute a cusp catastrophe model

B_2_ and C constitute a swallowtail catastrophe model

B_3_ and C constitute a cusp catastrophe model

#### Calculation of Comprehensive Membership Values of Chemical Enterprise Environmental Risks

A and B constitute a swallowtail catastrophe model
